# Prevalence and determinants of cognitive impairment in older adults with stroke in China: a systematic review

**DOI:** 10.3389/fpubh.2025.1706560

**Published:** 2025-12-10

**Authors:** Yang Lan, Xue Chen, Xinghua Lei

**Affiliations:** Department of Neurology, Affiliated hospital of North Sichuan Medical College, Nanchong, Sichuan, China

**Keywords:** stroke, cognitive impairment, older adults, prevalence, risk factors, meta-analysis

## Abstract

**Background:**

This systematic review aims to estimate the pooled prevalence of cognitive impairment among older adults Chinese stroke patients and to identify its demographic, clinical, and biochemical determinants, thereby providing evidence to support effective clinical prevention and intervention strategies.

**Methods:**

Eight databases (CNKI, VIP, WanFang, CBM, PubMed, Web of Science, Embase, and the Cochrane Library) were systematically searched from inception to October 24, 2024. Studies were included if they enrolled Chinese older adults stroke patients (aged ≥60 years), analyzed risk factors for cognitive impairment using case–control/cohort designs, and adopted validated cognitive assessment tools such as the Mini-Mental State Examination [MMSE], Montreal Cognitive Assessment [MoCA]). Data extraction and quality assessment (using the Newcastle–Ottawa Scale) were independently performed by two reviewers. Meta-analysis was performed using Stata version 17.0. A random-effects model was applied for high heterogeneity (I^2^ ≥ 50%), whereas a fixed-effects model was used otherwise. Publication bias was assessed using Egger’s test and trim-and-fill method.

**Results:**

A total of 46 studies, comprising 8,236 older adults stroke patients (3,281 with cognitive impairment) were included in the analysis. The pooled prevalence of cognitive impairment was 42.4% (95%CI: 36.6–48.3%), with significant heterogeneity (I^2^ = 97.1%). Subgroup analyses showed higher prevalence in northern China (45.1%) compared with southern China (41.0%) and higher detection rates when using the MoCA (50.5%) than the MMSE (43.4%). The meta-analysis identified 13 robust risk factors, including female gender (OR = 4.167), hypertension (OR = 2.824), diabetes mellitus (OR = 3.344), frontal/temporal lobe infarction (OR = 1.615/1.739), multiple cerebral infarctions (OR = 2.583), brain atrophy (OR = 2.943), hyperhomocysteinemia (OR = 3.043), high-sensitivity C-reactive protein (Hs-CRP) (OR = 4.331), and National Institutes of Health Stroke Scale (NIHSS) scores (OR = 1.977) (all *p* < 0.05). Publication bias was detected in age-related analyses, and sensitivity analysis confirmed result stability except for CRP.

**Conclusion:**

Cognitive impairment affects 42.4% of older adults Chinese stroke patients and associated with modifiable risk factors (e.g., hypertension, diabetes) and anatomical correlates (e.g., frontal/temporal infarction). Future research should prioritize large-scale, prospective cohort studies to validate these findings and develop targeted interventions.

## Introduction

China is experiencing a rapid aging population, with individuals aged 60 years and older accounting for 18.7% (264 million) of the total population, as per the 2020 Seventh National Population Census (1, 2). Stroke has emerged as the leading cause of death and disability among Chinese adults, characterized by high morbidity, disability, mortality, recurrence rates, and economic burden (3, 4). Notably, 50.81% of stroke cases in China involve individuals aged 60 and above, with the 60–69 age group representing the largest proportion (28.69%) (5). Post-stroke cognitive impairment (PSCI), defined as cognitive decline occurring within 6 months of a stroke event, has become a critical contributor to long-term disability and has reduced the quality of life for stroke survivors (6). Approximately 50% of stroke patients develop cognitive impairment within the first year post-stroke, underscoring the urgent need of addressing this issue.

The pathogenesis of PSCI is multifactorial, involving vascular, neuroanatomical, and inflammatory mechanisms. Previous studies have identified various risk factors, including demographic characteristics (e.g., female gender, advanced age), vascular comorbidities (e.g., hypertension, diabetes mellitus, hyperlipidemia), neuroimaging findings (e.g., frontal/temporal lobe infarction, multiple cerebral infarctions, brain atrophy), and biochemical markers (e.g., hyperhomocysteinemia, high-sensitivity C-reactive protein [Hs-CRP]) (7, 8). For instance, female gender has been associated with a 4.167-fold increased risk of PSCI, whereas hypertension and diabetes mellitus confer odds ratios (ORs) of 2.824 and 3.344, respectively.

However, the reported prevalence of cognitive impairment in older adults Chinese stroke patients varies widely across studies, ranging from 10.1 to 76.9%, which can be attributed to differences in study designs, diagnostic tools (e.g., Mini-Mental State Examination [MMSE] vs. Montreal Cognitive Assessment [MoCA]), and patient populations (9). This heterogeneity complicates efforts to establish standardized prevalence estimates and evidence-based preventive strategies. Furthermore, while some risk factors have been consistently identified, the strength of their associations and generalizability across different populations remain unclear (10).

This systematic review aims to synthesize existing evidence to: (1) estimate the pooled prevalence of cognitive impairment in older adults Chinese stroke patients; (2) identify robust demographic, clinical, and biochemical determinants; and (3) inform clinical practice and research priorities. By integrating data from 46 eligible studies, this review aims to provide a comprehensive understanding of the epidemiological landscape of PSCI in this vulnerable population, thereby facilitating the development of targeted interventions to mitigate cognitive decline following stroke.

## Methods

### Search strategy

Eight databases were systematically searched: CNKI, VIP, WanFang, CBM, PubMed, Web of Science, Embase, and the Cochrane Library, from their inception to October 24, 2024. The search combined Medical Subject Headings (MeSH) terms and free keywords, including Chinese, China, older adults, aged, middle age, risk factor, predicted factor, impact, reason, relevant factor, influencing factor, cognitive dysfunctions, cognitive impairment, cognitive disorder, mild cognitive impairment, cognitive decline, mental deterioration, hypertension, high blood pressure, etc. Taking PubMed as an example, the detailed search formula is shown in Table 1.

**Table 1:** Search strategies for Chinese and English databases.

Database	Search strategy
PubMed	((((“Stroke”[Mesh]) OR (Cerebrovascular Accident*)) OR (stroke*)) AND ((((“Cognitive Dysfunction”[Mesh]) OR (Cognitive defect*)) OR (Cognitive Dysfunction*))) AND ((Risk Factor) OR (Influencing Factor*)))
Cochrane Library	#1 (stroke*): ti, ab, kw OR (Cerebrovascular Accident*): ti, ab, kw #2 (Cognitive Dysfunction*): ti, ab, kw OR (Cognitive defect*): ti, ab, kw #3 (Risk Factor*): ti, ab, kw OR (Influencing Factor*): ti, ab, kw #4 #1 AND #2 AND #3
Web of Science	#1 TS = (stroke*) OR TS = (Cerebrovascular Accident*) #2 TS = (Cognitive Dysfunction*) OR TS = (Cognitive defect*) #3 (TS = (Risk Factor*)) OR TS = (Influential factor*) #4 #1 AND #2 AND #3
Embase	#1 ‘cerebrovascular accident’/exp. OR ‘stroke*’: ti, ab, kw #2 ‘cognitive defect’/exp. OR ‘cognitive dysfunction*’: ti, ab, kw #3 ‘risk factor*’: ti, ab, kw OR ‘influencing factor*’: ti, ab, kw #4 #1 AND #2 AND #3
CNKI (China National Knowledge Infrastructure)	(((((Subject % = ‘Stroke’ or Title % = ‘Stroke’ or title = xlsw (‘Stroke’) or v_subject = xlsw (‘Stroke’)) OR (Subject % = ‘Intracranial Embolism and Thrombosis’ or Title % = ‘Intracranial Embolism and Thrombosis’ or title = xlsw (‘Intracranial Embolism and Thrombosis’) or v_subject = xlsw (‘Intracranial Embolism and Thrombosis’))) OR (Subject % = ‘Intracerebral Hemorrhage’ or Title % = ‘Intracerebral Hemorrhage’ or title = xlsw (‘Intracerebral Hemorrhage’) or v_subject = xlsw (‘Intracerebral Hemorrhage’)))) AND (Subject % = ‘Cognitive Dysfunction’ or Title % = ‘Cognitive Dysfunction’ or title = xlsw (‘Cognitive Dysfunction’) or v_subject = xlsw (‘Cognitive Dysfunction’))) AND ((Subject % = ‘Influencing Factor’ or Title % = ‘Influencing Factor’ or title = xlsw (‘Influencing Factor’) or v_subject = xlsw (‘Influencing Factor’)) OR (Subject % = ‘Influencing Factor’ or Title % = ‘Influencing Factor’ or title = xlsw (‘Influencing Factor’) or v_subject = xlsw (‘Influencing Factor’))))
Wanfang Data Knowledge Service	Subject: (Stroke OR Intracranial Embolism and Thrombosis OR Intracerebral Hemorrhage) AND Subject: (Cognitive Dysfunction) AND Subject: (Influencing Factor OR Impact Factor)
CBM (China Biology Medicine disc)	(“Stroke”[Common Field: Intelligence] OR “Intracranial Embolism and Thrombosis”[Common Field: Intelligence] OR “Intracerebral Hemorrhage”[Common Field: Intelligence]) AND “Cognitive Dysfunction”[Common Field: Intelligence] AND (“Influencing Factor”[Common Field: Intelligence] OR “Influencing Factor”[Common Field: Intelligence])
VIP (VIP Chinese Journal Service Platform)	((((Title or Keyword = Stroke OR Title or Keyword = Intracranial Embolism and Thrombosis) OR Title or Keyword = Intracerebral Hemorrhage) AND Title or Keyword = Cognitive Dysfunction) AND (Title or Keyword = Influencing Factor OR Title or Keyword = Impact Factor))

Inclusion criteria: ① Studies on Chinese older adults (≥60 years) stroke patients (ischemic/hemorrhagic); ② Case–control/cohort designs analyzing risk factors for cognitive impairment; Use of validated cognitive assessment tools (e.g., MMSE, MoCA); ③ Full-text articles in Chinese/English with extractable data. Exclusion criteria: ① Non-Chinese/English publications or abstracts; ② Duplicate studies or those with incomplete data; ③ Reviews, meta-analyses, or studies without cognitive outcomes.

### Literature screening and data extraction

Two reviewers independently screened literature and extracted data using Rayyan software, with discrepancies resolved through consultation with a third reviewer. The extracted information included: ① First author, publication year, study region; ② Sample size (cases/controls), stroke subtype, cognitive assessment tool; ③ Reported risk factors (demographic, clinical, biochemical indices); ④ Adjusted ORs with 95% confidence intervals (Cis) and statistical significance.

### Quality assessment

Two researchers independently used the Newcastle–Ottawa Qualification Scale (NOS) to evaluate the quality of the included studies, and then cross-checked them. If the scoring results were different, they would seek third-party opinions before making a decision. The evaluation content of this scale includes three modules: study population selection (4 items, 4 points), inter-group comparability (1 item, 2 points), and exposure/outcome (3 items, 3 points), with a total score of 9 points. Studies were categorized as high (8, 9), moderate (5–7), or low (<5) quality based on their scores.

### Statistical analysis

Statistical analyses were performed using Stata 17.0 software. The ORs and 95% confidence intervals (CIs) of various factors were used as combined effect sizes. Heterogeneity among studies was evaluated by the *I*^2^ statistic and Cochran’s Q test. A fixed-effect model was adopted when *p* ≥ 0.1 and I^2^ < 50%, indicating small heterogeneity, while a random-effect model was used when *p* < 0.1 and I^2^ ≥ 50%, suggesting substantial heterogeneity. Sensitivity analysis was conducted by comparing the results of random-effect and fixed-effect models after excluding each study one by one to assess the stability of the pooled estimates. Publication bias was detected by Egger’s test, and statistical significance was set at *p* < 0.05.

## Results

### Literature retrieval and screening

**Figure 1:**
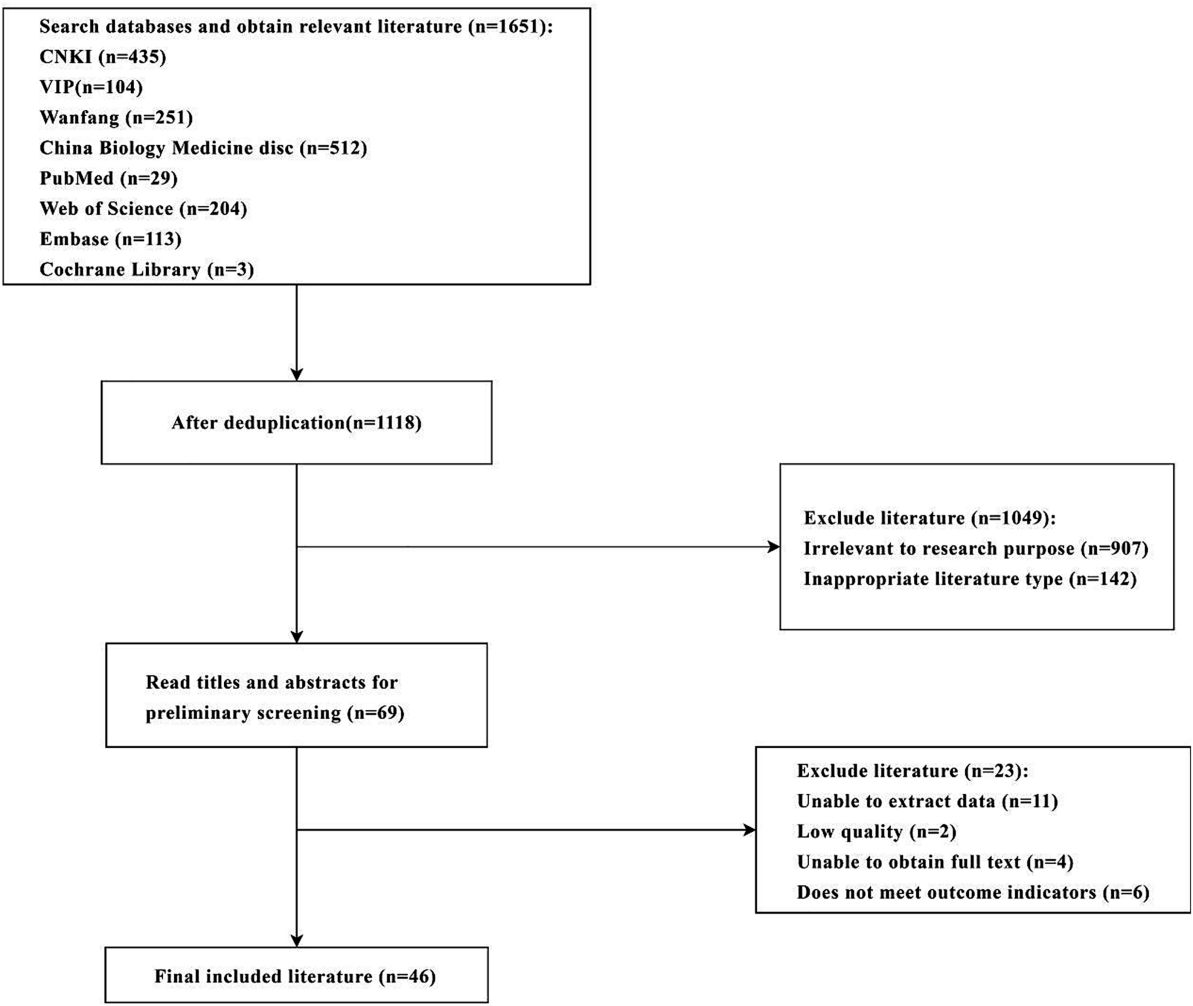
Flowchart of literature screening.

An initial search yielded 1,651 relevant articles. After removing duplicates, 1,118 articles remained. Through sequential screening by reading titles and abstracts, 46 articles (5, 10–54) met the inclusion criteria. The screening process and results are shown in Figure 1.

### Basic characteristics and quality assessment results of included literatures

A total of 46 publications were included, with an overall sample size of 8,236 cases, of whom 3,281 had cognitive impairment. Twenty-seven influencing factors, such as age, smoking, and drinking, were extracted and are shown in Table 2.

**Table 2:** Basic characteristics and quality assessment results of included studies.

Serial number	Included study	Year	Study type	Study region	Study subjects	Assessment tool	Age (years)	Number of cognitively impaired cases	Number of non - cognitively impaired cases	Prevalence (%)	Influencing factors	Cognitive assessment score (points)
1	Tang (9)	2006	Cross - sectional study	Hong Kong	Stroke patients	A	≥60	39	140	21.8	2, 3, 23	8
2	Wang Qingqing (10)	2010	Cross - sectional study	Zhengzhou	Ischemic stroke patients	A	≥60	30	711	10.1	8	5
3	Yang Hong (11)	2011	Cross - sectional study	Qinhuangdao	Cerebral infarction patients	A, D	≥60	57	56	50.4	3, 18	7
4	Zhang Zhijian (12)	2014	Cross - sectional study	Chongqing	Cerebral infarction patients	A, C	≥65	76	313	19.5	9, 18, 20, 22	5
5	Yuan Zhen (13)	2014	Cross - sectional study	Haikou	Lacunar infarction patients	B, D	≥60	68	82	45.3	1, 3, 10, 11, 16	8
6	Liu Ping (14)	2014	Cross - sectional study	Yangzhou	Ischemic stroke patients	B	≥60	52	50	51.0	1, 3, 10, 12, 13	5
7	Liu Tao (15)	2015	Cross - sectional study	Nanjing	Acute cerebral infarction patients	B	≥60	47	58	44.8	9, 10, 14, 20, 21, 23	6
8	Li Zhenghai (16)	2015	Cross - sectional study	Foshan	Lacunar infarction patients	B	≥60	52	50	51.0	10, 12, 13	7
9	Lei Jun (17)	2015	Cross - sectional study	Suining	Cerebral infarction patients	B, D	≥60	67	170	28.3	1, 4, 9, 10	7
10	Zhang Bo (18)	2017	Cross - sectional study	Zhoushan	Cerebral infarction patients	A, B	≥60	46	36	56.1	14	5
11	Li Haihua (19)	2017	Cross - sectional study	Qianxi	Lacunar infarction patients	B	≥60	41	78	34.5	1, 10, 14, 15, 16, 17	6
12	Xiao Mingyan (20)	2017	Cross - sectional study	Chongqing	First - ever cerebral infarction patients	A	≥70	159	141	53.0	3, 4, 5, 8, 23	9
13	Xie Chunqing (21)	2017	Cross - sectional study	Haikou	Acute ischemic stroke patients	A, F	≥80	201	225	47.2	6, 9, 12, 18	7
14	Yang Xiaowang (22)	2018	Case - control study	Yangzhou	Lacunar infarction patients	B	≥64	47	47	-	9, 10, 12, 13	7
15	Cheng Gaofei (23)	2018	Cross - sectional study	Neijiang	Lacunar infarction patients	B	≥60	64	53	54.7	9, 10	6
16	Yao Fan (24)	2018	Cross - sectional study	Chengdu	Ischemic stroke patients	A	≥60	90	127	41.5	1, 7	8
17	Zhang Xuehai (25)	2019	Cross - sectional study	Sanya	Acute cerebral infarction patients	A	≥62	77	106	42.1	9, 10, 13, 15, 23	7
18	Yang Yewen (26)	2019	Cross - sectional study	Foshan	Cerebral infarction patients	B	≥60	115	253	31.3	1, 2, 5, 9, 10, 20, 23	6
19	Wang Jing (27)	2019	Cross - sectional study	Nanchang	Cerebral infarction patients	B, D	≥60	82	41	66.6	1, 3, 9, 10	6
20	Wang Lan (28)	2019	Cross - sectional study	Yangzhou	Stroke patients	B	≥65	21	69	23.3	18	6
21	Chen Shujin (29)	2019	Cross - sectional study	Dongguan	Cerebral infarction patients	B	≥60	22	78	22.0	1, 17	6
22	Zhao Pei (30)	2020	Case - control study	Lianyungang	Ischemic stroke patients	A, B	>65	90	90	-	9	8
23	Zhang Hua (31)	2020	Cross - sectional study	Beijing	Ischemic stroke patients	B	≥65	67	49	57.8	1, 8, 20	7
24	Yang Huaqing (32)	2020	Cross - sectional study	Xinjiang	Ischemic stroke patients	A	≥62	51	69	42.5	1, 7	6
25	Sun Leshan (33)	2020	Cross - sectional study	Shanghai	Acute cerebral infarction patients	B	≥62	43	57	43.0	9, 10	6
26	Ma Mingjuan (34)	2020	Cross - sectional study	Luoyang	Acute cerebral infarction patients	B	≥60	43	69	38.3	1, 8	6
27	Liu Na (35)	2020	Cross - sectional study	Shenyang	Acute cerebral infarction patients	A	≥60	55	184	23.0	1, 9, 10, 14, 15	6
28	Li Xiangquan (36)	2020	Cross - sectional study	Xuzhou	Acute cerebral infarction patients	A	≥62	77	106	42.1	9, 10, 13, 15, 23	6
29	Xie Daijian (37)	2021	Cross - sectional study	Huizhou	First - ever ischemic stroke patients	B	≥61	57	123	31.7	-	5
30	Wang Ying (38)	2021	Cross - sectional study	Beijing	First - ever acute cerebral infarction patients	B	≥60	169	58	74.4	2, 3, 24	8
31	Wang Haiying (39)	2021	Cross - sectional study	Zhuhai	Acute ischemic stroke patients	-	≥60	106	194	35.3	3, 9, 10, 17, 21, 23	5
32	Shen Yang (40)	2021	Cross - sectional study	Nanjing	Acute cerebral infarction patients	B	≥65	71	54	56.8	11, 18	6
33	Lu Zhenhui (41)	2021	Cross - sectional study	Nantong	First - ever cerebral infarction patients	B	>60	163	107	60.4	3, 9, 18, 20	7
34	Huang Xiaoyun (42)	2021	Cross - sectional study	Dongguan	First - ever stroke patients	A, B	≥60	47	54	46.5	4, 9, 10, 12, 20, 21	7
35	Hao Chaowei (43)	2021	Cross - sectional study	Tianjin	Acute cerebral infarction patients	A, B	≥60	54	46	54.0	27	7
36	Feng Yujie (44)	2021	Cross - sectional study	Tangshan	Lacunar infarction patients	A, B, E	>65	117	151	43.7	2, 10, 21	7
37	Shan Na (45)	2021	Cross - sectional study	Qinhuangdao	Acute ischemic stroke patients	B	≥65	39	81	32.5	1, 2, 3, 9, 10, 18, 19, 21, 22	6
38	Zhou Fei (46)	2022	Cross - sectional study	Haikou	Ischemic stroke patients	A	≥60	33	59	35.9	1, 9	6
39	Yang Xiaolan (47)	2022	Cross - sectional study	Baiyin	Acute cerebral infarction patients	A	≥60	49	44	52.7	25	7
40	Wu Jiahong (48)	2022	Cross - sectional study	Zhangjiakou	Mild acute cerebral infarction patients	B	≥65	150	45	76.9	2, 3, 9, 10, 14, 24	6
41	Ni Huafu (49)	2022	Cross - sectional study	Zhejiang	Lacunar infarction patients	B	≥61	57	50	53.3	9, 10, 14, 18, 20	8
42	Luo Yanfang (50)	2022	Cross - sectional study	Wuxi	Ischemic stroke patients	B	≥60	61	75	44.9	1, 3, 4, 6, 9	6
43	Deng Xiaoying (51)	2022	Cross - sectional study	Guangzhou	First - ever acute mild ischemic stroke patients	B	≥63	17	45	37.8	1, 5, 10, 18	6
44	Chen Zhizhi (52)	2022	Cross - sectional study	Quzhou	Lacunar infarction patients	A	≥61	15	47	24.2	1, 3, 9	7
45	Chen Xia (53)	2022	Cross - sectional study	Yangzhou, Linyi	Acute mild stroke patients	A, G	≥60	77	144	34.8	1, 3, 17	7
46	Wang Jing (54)	2023	Case - control study	Sanya	Acute cerebral infarction patients	B	≥60	70	70	-	26	8

In the diagnostic criteria, A represents the Mini-Mental State Examination (MMSE) (63), B represents the Montreal Cognitive Assessment (MoCA) (64), C represents the criteria for Mild Cognitive Impairment (MCI) diagnosis (65), D represents the criteria for Vascular Cognitive Impairment (VIC) diagnosis (66–68), E represents the Cambridge Cognitive Examination for the older adults (CAMCOG) (68), F represents the guidelines for the diagnosis of vascular cognitive impairment (69), G represents the Informant Questionnaire on Cognitive Decline in the older adults (IQCODE) (70); Among the influencing factors, 1 = age, 2 = gender, 3 = years of education, 4 = smoking, 5 = drinking, 6 = physical exercise, 7 = frailty, 8 = depression, 9 = hypertension, 10 = diabetes, 11 = osteoporosis, 12 = hyperlipidemia, 13 = carotid plaque, 14 = location of infarction, 15 = multiple cerebral infarctions, 16 = large-area cerebral infarction, 17 = brain atrophy, 18 = homocysteine (Hcy), 19 = 25 - hydroxyvitamin D [25(OH)D], 20 = C-reactive protein (CRP), 21 = high-density lipoprotein cholesterol (HDL - C), 22 = low-density lipoprotein cholesterol (LDL - C), 23 = National Institutes of Health Stroke Scale (NIHSS) score, 24 = Health-Promoting Behaviors Scale for Stroke Patients (HBS - SP) ≤ 55 points, 25 = common intestinal bacteria, 26 = zonula occludens - 1 (ZO - 1), 27 = grading and quantity of collateral circulation establishment.

### Meta-analysis of the prevalence of cognitive impairment

#### Overall prevalence

**Figure 2:**
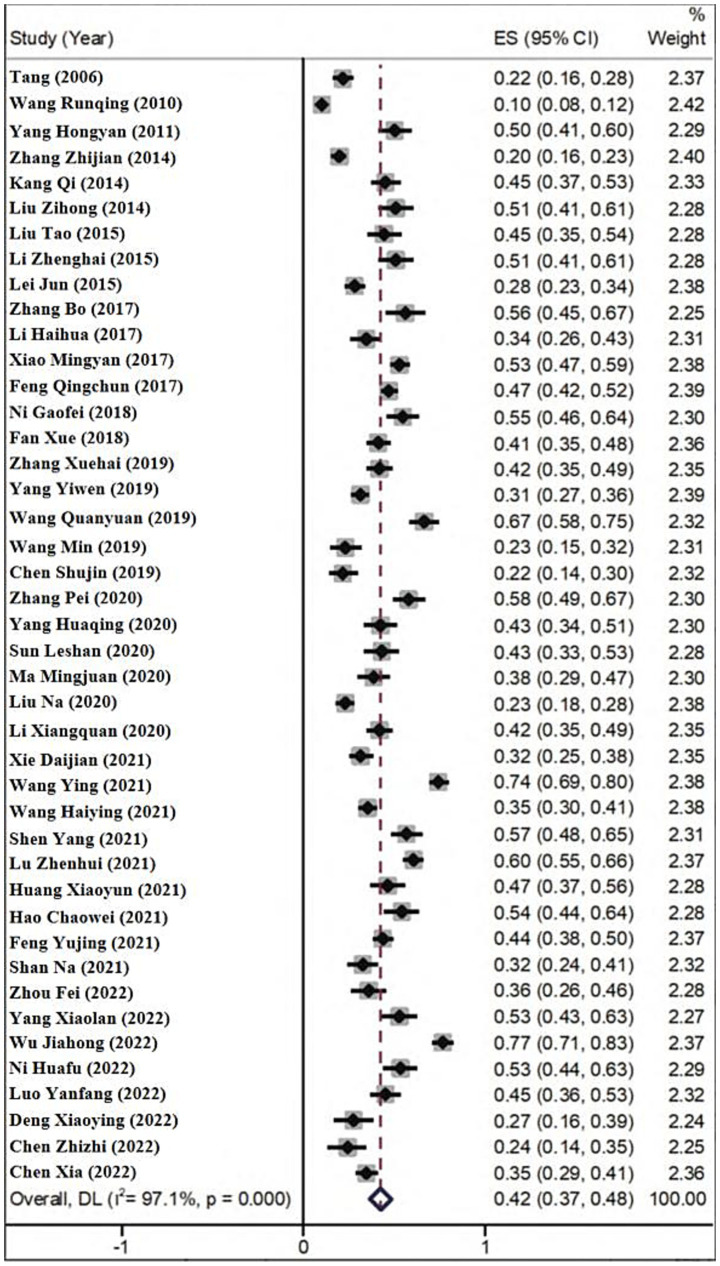
Prevalence of cognitive impairment in older adults stroke patients in China.

For the meta-analysis of the prevalence, 43 cross-sectional studies among the included literatures were analyzed. The results showed that *I*^2^ = 97.1% and *p* < 0.001. Therefore, the random-effects model was applied for pooling. The results indicated that the prevalence of cognitive impairment in older adults stroke patients in China was 42.4% [95%CI (36.6, 48.3%)], as shown in Figure 2.

#### Subgroup analysis

Subgroup analyses of the included studies were conducted by study location and diagnostic criteria. When grouped by study location, the prevalence of cognitive impairment in older adults stroke patients in the northern and southern regions was 45.1% [95%CI (31.9, 58.4%)] and 41.0% [95%CI (35.9, 46.0%)], respectively. When grouped by assessment tools, for older adults stroke patients assessed with the Mini-Mental State Examination (MMSE), Montreal Cognitive Assessment (MoCA), and MMSE + MoCA, the prevalence rates were 43.4% [95%CI (36.9, 49.8%)], 50.5% [95%CI (43.6, 57.4%)], and 47.9% [95%CI (43.8, 52.1%)], respectively, as shown in Table 3.

**Table 3:** Results of subgroup analysis of prevalence.

Subgroup	Number of literatures	Prevalence analysis (I^2^)/(%)	Model selection	95%CI (%)	*p*-value
Study Region					
Northern China	15 (10, 11, 14, 31, 32, 34–36, 38, 43–45, 47, 48)	98.6	①	45.1 (31.9, 58.4)	<0.001
Southern China	28 (9, 12, 13, 15, 16, 18–21, 23–29, 33, 37, 39–42, 46, 49–53)	92.8	①	41.0 (35.9, 46.0)	<0.001
Assessment Tool					
MMSE<27ⁿ	13 (18, 20, 25, 31, 35, 36, 42–44, 46, 47, 52, 53)	89.1	①	43.4 (36.9, 49.8)	<0.001
MoCA<26ⁿ	20 (14–18, 23, 27, 33, 34, 37, 38, 40–45, 48–50)	93.6	①	50.5 (43.6, 57.4)	<0.001
MMSE<27ⁿ + MoCA<26ⁿ	4 (18, 42–44)	48.0	②	47.9 (43.8, 52.1)	<0.001

In model selection, ① represents the random-effects model, ② represents the fixed-effects model; and ⁿindicates the cutoff values determined by the assessment tools.

#### Meta-regression and sensitivity analysis

Meta-regression analysis was performed on the prevalence of ≥ 10 included literatures (9–54). With publication year, study subjects, study location, and assessment tool as covariates for meta-regression analysis, no sources of heterogeneity were found. For the combined results with I^2^ > 50% and > 2 included literatures, sensitivity analysis was conducted by removing individual studies sequentially, and all the results showed that the research results were relatively stable.

#### Publication bias analysis

Egger’s test was used to test the publication bias of literatures with ≥ 10 included articles. A *p* < 0.05 suggests the existence of publication bias. The trim-and-fill method was used to evaluate the specific number of missing studies for the results with publication bias and to assess the degree of impact of publication bias on the results. Before and after trim and fill, the *p* values were < 0.05, and the combined results were stable as shown in Table 4.

**Table 4:** Analysis results of publication bias.

Item	Publication bias from Egger’s Test (*P*-value)	Trim-and-fill method				
		Number of trims	Effect size before trimming		Effect size after trimming	
			OR (95%CI)	*P*-value	OR (95%CI)	*P*-value
Total Prevalence	<0.001	20	0.424 (0.366, 0.483)	<0.001	1.312 (1.232, 1.398)	<0.001
Cerebral Infarction/Ischemic Stroke	<0.001	19	0.435 (0.372, 0.499)	<0.001	1.312 (1.225, 1.404)	<0.001
Northern Region	0.002	8	0.451 (0.319, 0.584)	<0.001	1.244 (1.083, 1.429)	0.002
Southern Region	0.098	–	–	–	–	–
MMSE	0.253	–	–	–	–	–
MoCA	0.469	–	–	–	–	–

### Meta-analysis of influencing factors for cognitive impairment

#### Influencing factors

Meta-analysis of influencing factors was conducted on 46 included studies. The results of the meta-analysis showed that 13 items, including female gender (OR = 4.167), hypertension (OR = 2.824), systolic blood pressure (OR = 1.572), diabetes mellitus (OR = 3.344), hyperlipidemia (OR = 2.228), carotid plaque (OR = 2.544), location of infarct (frontal lobe, temporal lobe) (frontal lobe: OR = 1.615; temporal lobe: OR = 1.739), multiple cerebral infarctions (OR = 2.583), brain atrophy (OR = 2.943), homocysteine (Hcy) (OR = 2.209), hyperhomocysteinemia (OR = 3.043), hs-CRP (OR = 4.331), and NIHSS Score (OR = 1.977), were influencing factors for cognitive impairment in older adults stroke patients in China (*p* < 0.05, Table 5).

**Table 5:** Results of meta-analysis of influencing factors.

Influencing factor	Number of literatures	Sample size (case)		Heterogeneity		Combined effect size		*P*
		Cognitive impairment	Non - cognitive impairment	*I*^2^(%)	Model selection	OR value	95% CI	
Age (years)	12 (24–26, 28–30, 32, 33, 35, 46–48, 64–69)	657	1,203	90.9	①	1.475	(1.262, 1.724)	<0.001
Gender								
Male	3 (26, 44, 46)	271	485	68.8	①	1.505	(0.884, 2.564)	0.132
Female	3 (26, 39, 44)	358	243	64.7	①	4.167	(1.937, 8.967)	<0.001
Years of Education	5 (16, 27, 34, 41, 50–52)	494	610	76.8	①	0.859	(0.722, 1.021)	0.085
Years of Education ≤ n Years	2 (29, 51)	183	338	16.6	②	2.233	(1.533, 3.252)	<0.001
Smoking	4 (29, 34, 41, 50)	416	481	76.8	①	1.336	(0.984, 1.814)	0.064
Alcohol Drinking	3 (29, 30, 51)	291	439	59.1	①	1.044	(0.551, 1.944)	0.891
Physical Exercise	2 (29, 51)	262	300	12.9	②	0.704	(0.587, 0.845)	<0.001
Frailty	2 (29, 32)	141	196	0	②	8.908	(5.346, 14.843)	<0.001
Depression	2 (19, 29)	239	852	90.6	①	3.931	(0.863, 17.911)	0.077
Hypertension	20 (15, 17, 23–25, 27–31, 33–36, 39, 41, 48–50)	1,440	1825	55	①	2.824	(2.292, 3.481)	<0.001
Systolic Blood Pressure	3 (32, 33, 48)	324	592	14.4	②	1.572	(1.444, 1.711)	<0.001
Diabetes Mellitus	21 (14–17, 19–24, 26, 27, 31, 33–36, 39, 41, 48–50)	1,420	1955	76.1	①	3.344	(2.611, 4.284)	<0.001
Osteoporosis	2 (14, 30)	139	136	0	②	2.108	(1.411, 3.151)	<0.001
Hyperlipidemia	5 (14, 19, 28–32)	399	426	76.6	①	2.228	(1.091, 4.547)	0.028
Carotid Plaque	5 (8, 14, 18, 28, 36)	305	359	83.3	①	2.544	(1.707, 3.614)	0.033
Infarct Location								
Frontal Lobe	3 (15, 18, 48)	243	139	0	②	1.615	(1.167, 2.235)	0.004
Temporal Lobe	3 (15, 18, 48)	243	139	55.3	①	1.739	(1.246, 2.427)	0.001
Parietal Lobe	2 (18, 48)	196	81	0	②	1.445	(1.066, 2.157)	0.022
Occipital Lobe	2 (18, 48)	93	81	43.6	②	1.716	(1.125, 2.613)	0.132
Thalamus	2 (15, 18)	196	94	0	②	1.567	(1.035, 2.628)	0.035
Left Cerebral Hemisphere	3 (19, 25, 48)	153	312	78.5	①	2.915	(1.657, 5.138)	<0.001
Multiple Cerebral Infarctions	4 (14, 23, 38, 50)	250	474	40.3	②	2.583	(2.009, 3.320)	0.001
Large-area Infarction	2 (13, 19)	109	160	0	②	2.881	(1.560, 5.322)	0.001
Brain Atrophy	4 (19, 28, 30, 63)	246	494	0	②	2.943	(1.938, 4.468)	<0.001
Hcy	8 (14–17, 21, 33, 44, 48, 63)	573	767	91.5	①	2.209	(1.692, 2.949)	<0.001
Hyperhomocysteinemia	3 (21, 28, 31)	239	339	68	①	3.043	(2.092, 4.426)	<0.001
25(OH)D	3 (39, 41, 49)	317	441	93.9	①	1.270	(0.501, 3.221)	0.615
CRP	3 (33, 35, 48)	200	412	91	①	1.775	(1.164, 2.706)	0.008
Hs-CRP	3 (33, 42, 48)	207	262	80.5	①	4.331	(1.756, 10.685)	0.001
HDL-C	2 (24, 48)	86	135	66.5	①	1.189	(0.246, 5.696)	0.871
LDL-C	2 (24, 48)	115	394	0	②	1.309	(1.153, 1.963)	0.001
NIHSS	3 (36, 38, 64)	313	534	50.4	①	1.977	(1.320, 2.961)	0.001
NIHSS Score > 10 Points	2 (38, 54)	154	212	0	②	2.484	(1.800, 3.428)	<0.001
HBS-SP Score ≤ 55 Points	2 (34, 48)	319	103	0	②	2.454	(1.681, 3.583)	<0.001

In model selection, ① represents the random - effects model, ② represents the fixed - effects model.

#### Meta-regression and sensitivity analysis

A meta-regression analysis was conducted to explore the influencing factors of ≥ 10 included literatures (13–17, 19, 22–27, 29–36, 39, 41, 42, 44–46, 48–53). With publication year, study subjects, study location, and assessment tool being used as covariates for meta-regression analysis. Except for diabetes mellitus, no sources of heterogeneity were found for other influencing factors. For the combined results with I^2^ > 50%, sensitivity analysis was conducted, and the combined effect size was verified after removing individual studies one by one. Except for the unstable results when removing the studies by Zhang et al. (12) (*p* = 0.205) and Ni et al. (49) (*p* = 0.229) in CRP, all other meta-analyses showed that the research results were relatively stable. When the influencing factor was diabetes mellitus, through meta-regression analysis, it was found that the study subjects being older adults cerebral infarction patients [Coef. = 1.969, SE = 0.722, *p* = 0.013, 95%CI (0.458, 3.480)] and the assessment tool being MoCA [Coef. = 1.212, SE = 0.504, *p* = 0.027, 95%CI (0.155, 2.270)] were sources of heterogeneity (*p* < 0.05). Among the combined results where diabetes mellitus was an influencing factor, it might be because among the 21 included studies, the study subjects of Huang et al. (42) were first-ever stroke patients, and the study subjects of the remaining 20 articles were cerebral infarction patients. “First-ever stroke” encompasses a patient’s first acute cerebrovascular event, including both ischemic stroke (cerebral infarction) and hemorrhagic stroke. In contrast, “cerebral infarction” specifically refers to the ischemic stroke subtype. Consequently, the study by Huang et al. included a broader population of stroke patients, while the other studies focused specifically on those with cerebral infarction; 16 studies used MoCA as the assessment tool, 3 studies used MMSE, and 2 studies used MMSE + MoCA. Sensitivity analysis, performed by removing studies sequentially, showed that the result that diabetes mellitus was an influencing factor for cognitive impairment in older adults Chinese stroke patients was stable.

### Publication bias analysis

Egger’s test was used to test the publication bias of literatures with ≥ 10 included articles. A *p* value < 0.05 suggests the existence of publication bias. The trim-and-fill method was applied to evaluate the degree of impact of publication bias on the results for the results with publication bias. The OR value of age (years) was < 1 before trim and fill and > 1 after trim and fill, suggesting that publication bias had a relatively large impact on the results, and the results were unstable, as shown in Table 6.

**Table 6:** Analysis results of publication bias.

Influencing factor	Publication bias from Egger’s test (*P*-value)	Trim-and-fill method	Effect size before trimming		Effect size after trimming	
		Number of trims	OR (95%CI)	*P*-value	OR (95%CI)	*P*-value
Age (years)	0.012	6	0.389 (0.233, 0.545)	<0.001	1.226 (1.049, 1.431)	0.010
Hypertension	0.012	5	1.038 (0.829, 1.247)	<0.001	2.379 (1.878, 3.014)	<0.001
Diabetes Mellitus	0.186	5	1.207 (0.960, 1.455)	<0.001	2.769 (2.157, 3.554)	<0.001

### Descriptive analysis

Descriptive analysis was conducted on the research results that could not be subjected to combined effect size meta-analysis among the 46 included studies on influencing factors. The study by Yang et al. (47) showed that Enterobacter [OR = 1.777, 95% CI (1.064, 2.966)] and Enterococcus [OR = 1.689, 95%CI (1.088, 2.624)] were influencing factors for the occurrence of cognitive impairment in older adults patients with acute cerebral infarction (*p* < 0.05); Bifidobacterium [OR = 0.213, 95%CI (0.085, 0.532)], Lactobacillus [OR = 0.242, 95%CI (0.097, 0.603)], and Peptostreptococcus [OR = 0.409, 95%CI (0.203, 0.826)] were protective factors for the occurrence of cognitive impairment (p < 0.05). Occludin and ZO-1 are integral components of tight junctions, which play a crucial role in maintaining the integrity of the blood–brain barrier (BBB). Occludin regulates the permeability of the BBB, whereas ZO-1 interacts with other tight junction proteins to stabilize the BBB and prevent harmful substances from entering the brain. Disruption of these proteins has been associated with increased BBB permeability, which can lead to neuroinflammation and cognitive impairment. These findings highlight the potential role of BBB disruption in cognitive decline following acute cerebral infarction. The study by Wang et al. (54) showed that serum occludin [OR = 2.721, 95%CI (1.100, 6.730)] and zonula occludens-1 (ZO - 1) [OR = 2.824, 95%CI (1.162, 6.862)] were influencing factors for the occurrence of cognitive impairment in older adults patients with acute cerebral infarction (*p* < 0.05). And these bacteria were identified in the gut microbiome of the patients, and their association with cognitive decline may be linked with the gut–brain axis, where gut microbiota potentially influence cognitive function through immune modulation, neurochemical changes, or blood–brain barrier permeability. The study by Hao et al. (43) showed that the grading of collateral circulation establishment [OR = 4.809, 95%CI (0.319, 72.558)] and the quantity of collateral circulation establishment [OR = 1.243, 95%CI (0.005, 283.568)] were influencing factors for the occurrence of cognitive impairment in older adults patients with acute cerebral infarction (p < 0.05).

## Discussion

The pooled prevalence of cognitive impairment among Chinese older adults stroke patients was 42.4% [95%CI (36.6, 48.3%)], which is relatively high and higher than that in the UK (22%) (55). This study found: (1) The prevalence of cognitive impairment in northern China is higher than that in southern China. This may be related to differences in living habits, economic development, and the level of health information received by the population between the north and the south. (2) The prevalence of cognitive impairment detected by the Montreal Cognitive Assessment (MoCA) is higher than that by the Mini-Mental State Examination (MMSE). A meta-analysis showed that MoCA is superior to MMSE in screening for mild cognitive impairment in the older adults (56). MoCA is a 30-point cognitive screening tool that assesses various cognitive domains, including executive function, attention, memory, and language. In contrast, MMSE, while widely used, has a ceiling effect, making it less sensitive to early or subtle cognitive impairments. Moreover, the performance on both MoCA and MMSE can be influenced by the individual’s educational background. Pinto et al. (56) accounted for participants’ education level in their analysis, ensuring that the observed differences in sensitivity were not confounded by this factor. The MoCA is preferred for the clinical screening for cognitive impairment in older stroke patients. Among the articles included in the prevalence analysis, the study subjects included older adults patients with the following types of cerebral infarction: Mild cerebral infarction: A type of ischemic stroke characterized by a small, often clinically subtle, infarct in the brain, usually diagnosed based on imaging studies; First-ever cerebral infarction: This refers to the patients who experience their first ischemic stroke event, with no prior history of stroke or transient ischemic attacks; Acute cerebral infarction: A stroke that occurs within a short period, typically defined as occurring within 24–72 h of symptom onset, characterized by the sudden loss of brain function due to blockage of blood flow to the brain; Lacunar cerebral infarction: A subtype of ischemic stroke, which involves small infarcts (usually less than 1.5 cm in diameter) located in deep brain structures. The prevalence of mild cerebral infarction in the older adults is as high as 76.9% (48), probably because mild cerebral infarction has mild symptoms, patients have no obvious symptoms, and some patients only show mild mental function deficits such as inattention and memory decline, with a low risk of disability and good prognosis. Many older adults patients with cerebral infarction and their families may not fully recognize the early signs of cognitive decline or the importance of timely medical intervention. As a result, they may not seek or receive appropriate treatment in the early stages, which can lead to the progression of cognitive impairment over time. The prevalence of first-ever cerebral infarction, acute cerebral infarction, and lacunar cerebral infarction in the older adults is as high as 60.4% (41), 56.8% (40), and 54.7% (23). Patients with first-ever cerebral infarction and acute cerebral infarction typically present with acute onset, meaning that symptoms appear suddenly, often within a few hours to days. These patients may experience severe symptoms, such as pronounced neurological deficits (e.g., hemiparesis, aphasia, or loss of coordination), which indicate significant brain dysfunction. Furthermore, they often exhibit poor treatment compliance, which refers to the tendency to delay or not follow prescribed treatments or rehabilitation plans, particularly during the early phase of illness. As a result, early symptoms of cognitive impairment, such as mild memory deficits or difficulties with concentration, are often subtle and easily overlooked in clinical practice, especially during the acute phase of stroke. If not identified and addressed promptly, these symptoms can progress over time, leading to more severe cognitive impairment. (57). The prevalence of the above-mentioned patients is higher than the pooled prevalence of cognitive impairment in older adults stroke patients. Medical staff should be vigilant about the early signs of cognitive impairment in patients with acute or first-ever cerebral infarction, including symptoms such as mild memory loss, difficulty concentrating, confusion, trouble with planning or decision-making, and decreased attention span. Timely intervention, such as cognitive screening using tools like MoCA, can help identify these early signs. Additionally, healthcare providers should encourage early rehabilitation, including cognitive exercises and social engagement, and address any modifiable risk factors such as depression or sleep disturbances, which can exacerbate cognitive decline. Early treatment, along with education for patients and families about the importance of regular follow-up, can help prevent further deterioration of cognitive function.

Female gender, hypertension, systolic blood pressure, diabetes mellitus, hyperlipidemia, carotid plaque, location of infarct (frontal lobe and temporal lobe), multiple cerebral infarctions, brain atrophy, homocysteine (Hcy), hyperhomocysteinemia, high-sensitivity C-reactive protein (Hs-CRP), and National Institutes of Health Stroke Scale (NIHSS) score are the influencing factors for cognitive impairment in older adults stroke patients in China. Most female patients over 60 years old are in menopause, and the synthesis and secretion of estrogen by the ovaries decrease. The decrease in estradiol level leads to a decline in cognitive ability through the interaction of the cholinergic system, dopamine system, and mitochondrial bioenergetic system in the basal forebrain (58). Older adults patients with hypertension and high systolic blood pressure have a decrease in vascular autoregulation ability, which affects the blood supply and oxygen supply to brain tissue, increases the risk of white matter lesions around the ventricles and in the subcortex, and decreases brain metabolic ability, making them more prone to cognitive impairment (59). Diabetes mellitus can contribute to cognitive impairment through multiple mechanisms. In addition to causing dopamine dysfunction, which impairs behavioral and motor regulation, diabetes is associated with glucose fluctuations, the accumulation of advanced glycation end products (AGEs), and brain insulin resistance. These factors together disrupt normal brain function, impair cognitive abilities, and increase the risk of neurodegenerative changes. (60). Hyperlipidemia causes atherosclerosis, leading to vascular stenosis, affecting cerebral perfusion, and promoting the formation of neurofibrillary tangles and amyloid proteins in brain tissue (61). Unstable plaques are prone to rupture, blocking brain blood vessels, causing brain ischemia and hypoxia, leading to brain tissue damage, which impairs cognitive function damage. The frontal cortex is responsible for executive functions, with the lateral prefrontal cortex serving as the central hub. Patients with frontal lobe brain damage, especially left dorsal frontal lobe damage, have significantly impaired organizational ability, decreased ability to process and solve problems, and difficulty in organizing and executing plans (62). Damage to the temporal lobe, particularly the hippocampus, often results in significant memory impairments (delayed recall), orientation, abstraction ability, and calculation ability (62). A study showed that through the data of 2,950 stroke patients including cases with infarctions in various brain regions, found that left frontotemporal lobe infarction is closely related to the occurrence and development of post-stroke cognitive impairment (6). Patients with large-area cerebral infarction and brain atrophy have greater damage to the cerebrovascular system and a larger number of damaged brain cells, causing damage to the central nervous system of the patient’s brain. Hcy leads to impaired neuron function through mechanisms such as excitotoxicity, oxidative stress, blood vessel damage, and inhibition of methylation. Hs-CRP is a non-specific inflammatory protein and a sensitive marker of the inflammatory response. The increase in serum Hs-CRP level can lead to intracranial endothelial dysfunction and damage to the vascular integrity of the frontal cortex–subcortical loop, inhibit the formation of new blood vessels, and increase the degree of cerebrovascular damage. The NIHSS score is an important evaluation index for nervous system injury. The higher the score, the more severe the brain nerve damage of the patient will be, and the worse the cognitive function.

While this systematic review provides a comprehensive assessment of the prevalence and influencing factors of cognitive impairment in older adults Chinese stroke patients, several limitations should be acknowledged. First, a key methodological challenge across the included studies is the inability to reliably distinguish between pre-existing cognitive impairment present at the time of the stroke and genuine post-stroke cognitive deterioration. This distinction is vital for accurately attributing cognitive deficits to the stroke event itself. However, obtaining detailed pre-stroke cognitive baselines is often impractical in clinical and research settings, leading to a potential overestimation of the true incidence of post-stroke cognitive impairment (PSCI). This limitation is inherent to the design of most available studies and should be considered when interpreting the pooled prevalence. Secondly, while this review identifies modifiable vascular risk factors such as hypertension and diabetes as significant determinants, a critical gap in the available literature is the lack of information regarding the treatment status and management quality of these conditions. The included studies did not provide stratified data comparing patients with treated versus untreated conditions or with adequate versus inadequate risk factor control. Consequently, our analysis confirms the association between these comorbidities and cognitive impairment but cannot elucidate whether effective medical treatment modifies this risk, which is a crucial question for clinical prevention. Furthermore, in the analysis of influencing factors, publication bias was detected for age, and the sensitivity analysis for CRP indicated that the pooled result was unstable. More importantly, the pooled results for the following factors—education years < 6 years, physical exercise, frailty, osteoporosis, LDL-C, infarct location (occipital lobe, left cerebral hemisphere), large-area cerebral infarction, NIHSS score > 10 points, and HBS-SP score ≤ 55 points—were unstable due to the limited number of included studies (all ≤ 2), requiring verification through future research with larger sample sizes. Furthermore, the heterogeneity observed among the included studies may also influence the interpretation of some pooled estimates.

## Conclusion

In conclusion, this study shows that the prevalence of cognitive impairment in older adults stroke patients in China is 42.4%. Female gender, hypertension, systolic blood pressure, diabetes mellitus, hyperlipidemia, carotid plaque, location of infarct (frontal lobe, temporal lobe), multiple cerebral infarctions, brain atrophy, Hcy, hyperhomocysteinemia, Hs-CRP, and NIHSS score are the influencing factors for cognitive impairment in Chinese older adults stroke patients. In the prevalence study, due to considerable heterogeneity among the study populations included in the prevalence analysis, subgroup analysis by subject type could not be conducted. In the future, the prevalence can be refined and compared for a specific study subject. Most of the studies included in the influencing factor analysis are cross-sectional studies, and there is a lack of prospective studies, resulting in poor argumentation strength. Therefore, multi-center, large-sample, and high-quality cohort studies are needed to discuss the influencing factors of cognitive impairment in older adults stroke patients in China.

## Data Availability

The original contributions presented in the study are included in the article/supplementary material, further inquiries can be directed to the corresponding author.
